# Validation of Simultaneous Quantitative Method of HIV Protease Inhibitors Atazanavir, Darunavir and Ritonavir in Human Plasma by UPLC-MS/MS

**DOI:** 10.1155/2014/482693

**Published:** 2014-01-23

**Authors:** Tulsidas Mishra, Pranav S. Shrivastav

**Affiliations:** ^1^Chemistry Department, Kadi Sarva Vishwavidyalaya, Gandhinagar 382015, India; ^2^Department of Chemistry, School of Sciences, Gujarat University, Ahmedabad 380009, India

## Abstract

*Objectives*. HIV protease inhibitors are used in the treatment of patients suffering from AIDS and they act at the final stage of viral replication by interfering with the HIV protease enzyme. The paper describes a selective, sensitive, and robust method for simultaneous determination of three protease inhibitors atazanavir, darunavir and ritonavir in human plasma by ultra performance liquid chromatography-tandem mass spectrometry. *Materials and Methods*. The sample pretreatment consisted of solid phase extraction of analytes and their deuterated analogs as internal standards from 50 **μ**L human plasma. Chromatographic separation of analytes was performed on Waters Acquity UPLC C18 (50 × 2.1 mm, 1.7 **μ**m) column under gradient conditions using 10 mM ammonium formate, pH 4.0, and acetonitrile as the mobile phase. *Results*. The method was established over a concentration range of 5.0–6000 ng/mL for atazanavir, 5.0–5000 ng/mL for darunavir and 1.0–500 ng/mL for ritonavir. Accuracy, precision, matrix effect, recovery, and stability of the analytes were evaluated as per US FDA guidelines. *Conclusions*. The efficiency of sample preparation, short analysis time, and high selectivity permit simultaneous estimation of these inhibitors. The validated method can be useful in determining plasma concentration of these protease inhibitors for therapeutic drug monitoring and in high throughput clinical studies.

## 1. Introduction

Ever since the introduction of protease inhibitors (PIs) in 1995 for the treatment of human immunodeficiency virus (HIV) and their subsequent relevance in highly active antiretroviral therapy (HAART), there is an increase in the life expectancy of HIV infected patients and thus reduction in mortality of infected patients [1]. The HAART therapy relies on combination of several drugs in a daily regimen which includes one or more nucleoside reverse transcriptase inhibitors (NRTIs), together with one or two PIs and one NNRTI. This combination helps in minimizing occurrence of viral resistance and in preventing adverse events [2]. Standard guidelines recommend that initial treatment of patients with HIV-1 infection under HAART therapy should have a ritonavir- (RTV-) boosted protease inhibitor, usually darunavir (DRV) or atazanavir (ATV) together with other antiretrovirals (ARVs) [3]. Due to rapid emergence of resistance for NNRTIs, the use of PIs has increased for the treatment of HIV infection. PIs mainly affect the aspartic protease enzyme of human immunodeficiency virus (HIV PR) which is responsible for the cleavage of the viral Gag and Gag-Pol polyprotein precursors into mature, functional viral enzymes, and structural proteins [4].

RTV, a first-generation highly potent PI was designed by Abbot Laboratories and was approved by US FDA in 1996. It is active against HIV-1 as well as HIV-2 proteases and is currently used as a booster to optimize pharmacokinetics of other PIs and to prolong their therapeutic effects. It is sold under the brand name Norvir [5]. The second-generation PIs like ATV and DRV are used to inhibit those protease species which are resistant to the inhibitors of the first generation. Further, both the drugs are well tolerated with minimum side effects. ATV, an azapeptide, was developed originally by Ciba-Geigy and sold under the trade name Reyataz by Bristol-Myers Squibb. It was approved in 2003, and it shows a unique HIV resistance profile and favourable pharmacokinetics which allows once-daily dosing [6]. DRV is a nonpeptidic HIV PI, developed by Tibotec BVBA and approved by US FDA in 2006, and is commercially available as Prezista [7]. It has broad specificity against mutated and highly resistant protease species mainly due to its ability to fit to the proposed “substrate envelope” within the active site [1]. DRV is 100 times more effective for wild-type HIV-1 protease compared to several other PIs [8].

Accurate determination of PIs plasma concentration is vital for pharmacokinetic measurements, optimization of dosages, and drug-drug interaction studies. In spite of several clinical advantages of these PIs, they have a very narrow therapeutic index and hence the need for therapeutic drug monitoring is essential [9]. The literature presents several methods to determine ATV [10–13], DRV [14–17] and RTV [18, 19] as a single analyte using ELISA, HPLC-UV, LC-MS/MS, and UPLC-MS/MS techniques. Checa et al. [2] have reviewed methods for determination of antiretroviral drugs with special emphasis on the principal analytical strategies for dealing with clinical samples up to 2008. Since then there are numerous other methods which describe simultaneous determination of these drugs together with other PIs and antiretroviral drugs [20–31] in diverse matrices like human plasma [20–26], human whole blood and dried blood spots [27, 28], peripheral blood mononuclear cells [29, 30], and mouse serum and tissues [31]. Recently, an excellent review article has also been reported on bioanalytical methods developed for ARVs in tissues and different body fluids like amniotic fluid, cervicovaginal fluid, cerebrospinal fluid, extracellular cerebral fluid, saliva, and male seminal plasma/serum [32]. The bulk of these methods have employed LC-MS/MS [20, 22–25, 27–30] technique for the simultaneous analysis of ARVs, while the use of UPLC-MS/MS methodology for analysis has been a subject of very few reports [21, 26, 31]. Yadav et al. [21] analyzed lopinavir (LPV) and RTV in human plasma by UPLC-ESI-MS/MS and studied its application for a bioequivalence study in healthy subjects. In another report, three protease inhibitors indinavir (IDV), LPV, and RTV were determined simultaneously by UPLC-MS/MS [26]. Huang et al. [31] quantified nanoformulated RTV, IDV, ATV, and efavirenz in mouse serum and tissues by UPLC-MS/MS. However, there is no UPLC-MS/MS method for simultaneous determination of ATV, DRV and RTV in human plasma.

Ultra performance liquid chromatography (UPLC) has given a different dimension to separation science by building on the established principles of liquid chromatography. It functions on the use of sub, 2 *μ* particle size to provide increased resolution, sensitivity, and throughput. UPLC can reduce the analysis time and improve chromatographic performance compared to HPLC by controlling system volumes and peak dispersion. Additionally, solvent consumption can also be minimized compared to conventional 4.6 mm id columns [33]. Thus, in the present work, a robust, selective, and rapid UPLC-MS/MS method has been developed and fully validated for reliable measurement of ATV, DRV, and RTV in human plasma. The method employs only 50 *μ*L plasma volume for sample preparation and demonstrates excellent chromatographic efficiency (2.0 min). It can be readily applied in a high throughput clinical setting and also for therapeutic drug monitoring.

## 2. Materials and Methods

### 2.1. Chemicals

Reference standards of atazanavir (99.6%), darunavir (99.2%), and ritonavir (99.3%) and their deuterated internal standards (ISs) atazanavir-d6 (99.1%), darunavir-d9 (99.5%) and ritonavir-d6 (99.0%) were procured from Clearsynth Labs Pvt. Ltd. (Mumbai, India). HPLC grade methanol and acetonitrile were obtained from Mallinckrodt Baker, S.A.de C.V. (Estado de Mexico, Mexico). Bio-ultragrade ammonium formate and LC-MS grade formic acid were purchased from Sigma-Aldrich (St. Louis, MO, USA). Oasis HLB (1 cc, 30 mg) extraction cartridges were from Waters Corporation (Milford, MA, USA). Water used in the study was prepared from Milli-Q water purification system from Millipore (Bangalore, India). Blank human plasma in K_3_EDTA was obtained from Supratech Micropath (Ahmedabad, India) and was stored at –20°C until use.

### 2.2. Liquid Chromatography and Mass Spectrometric Conditions

The chromatographic analysis of ATV, DRV, and RTV was carried out on Waters Acquity UPLC system (MA, USA) employing BEH C18 (50 × 2.1 mm, 1.7 *μ*m) analytical column, maintained at 35°C. Separation was achieved under a gradient program using a mobile phase consisting of (A) 10 mM ammonium formate, pH 4.0, adjusted with formic acid in water, and (B) acetonitrile at a flow rate of 0.300 mL/min with 50% flow splitting. Initially, for up to 0.8 min, the ratio of A and B was kept at 50 : 50 (*v/v*) and from 0.8 min to 1.2 min the ratio was changed to 30 : 70 (*v/v*). The system was then equilibrated to the initial conditions up to 2.0 min. The sample manager temperature was maintained at 5°C with an alarm band of ±3°C and the average pressure of the system was 6000 psi.

Detection and quantitation of analytes and ISs were carried out using multiple reaction monitoring (MRM) for protonated precursor → product ion transitions on Quattro Premier XE mass spectrometer from Waters-Micro Mass Technologies (MA, USA) in the positive electrospray ionization mode. Source dependent and compound dependent mass parameters optimized and MRM transitions for analytes and ISs are summarized in Table 1. MassLynx software version 4.1 was used to control all parameters of UPLC and MS.

### 2.3. Standard Stock, Calibration Standards, and Quality Control Sample Preparation

The standard stock solutions of ATV, DRV, and RTV (1.0 mg/mL each) were prepared by dissolving requisite amounts in methanol. Their intermediate stock solutions and working solutions were made by appropriate dilution of their stock solutions with methanol : water (50 : 50, *v/v*). Calibration standards (CSs) and quality control (QC) samples were made by spiking blank plasma with appropriate volumes of working solutions. The concentration of CSs was 5.0, 10, 50, 100, 200, 400, 750, 1500, 3000, and 6000 ng/mL for ATV, 5.0, 10, 50, 100, 200, 400, 800, 1200, 2500, and 5000 ng/mL for DRV, and 1.0, 2.0, 5.0, 10, 20, 40, 80, 125, 250 and 500 ng/mL for RTV. The QC samples were prepared at five concentration levels as follows: HQC, high quality control: ATV (4800 ng/mL), DRV (4000 ng/mL), and RTV (400 ng/mL); MQC-1, medium quality control-1: ATV (2400 ng/mL), DRV (2000 ng/mL), and RTV (200 ng/mL); MQC-2, medium quality control-2: ATV (150 ng/mL), DRV (150 ng/mL), and RTV (30 ng/mL); LQC, low quality control: ATV (15 ng/mL), DRV (15 ng/mL) and RTV (3.0 ng/mL); and LLOQ QC, lower limit of quantification quality control: ATV (5.0 ng/mL), DRV (5.0 ng/mL) and RTV (1.0 ng/mL). The stock solutions of ISs (1.0 mg/mL) were prepared by dissolving 10.0 mg of ISs in 10.0 mL of methanol. Their working solution (500 ng/mL for ATV and DRV; 50 ng/mL for RTV) was prepared by appropriate dilution of the stock solution in methanol : water (50 : 50 *v/v*). The stock solutions were stored at 5°C, while calibration standards and quality control samples were stored at –70°C until use.

### 2.4. Sample Extraction Protocols

Prior to analysis, all calibration and quality control samples were thawed and allowed to equilibrate at room temperature. To an aliquot of 50 *μ*L of spiked plasma sample, 50 *μ*L internal standard was added and vortexed for approximately 10 s. Further, 100 *μ*L of 0.1% formic acid was added and vortexd for another 10 s. The samples were then loaded on Oasis HLB extraction cartridges which were preconditioned with 1 mL methanol followed by 1 mL of water. Thereafter the cartridges were washed with 1 mL, 5% methanol in water, and then dried for 2 min by applying nitrogen (1.72  ×  10^5^ Pa) at 2.4 L/min flow rate. Elution of analytes and ISs from the cartridges was carried out with 500 *μ*L of 0.2% formic acid in methanol into prelabeled tubes. The eluate was evaporated to dryness in a thermostatically controlled water-bath maintained at 40°C under a gentle stream of nitrogen for 5 min. After drying, the residue was reconstituted in 200 *μ*L of reconstitution solution (10 mM ammonium formate: acetonitrile (20 : 80, *v/v*)) and 5 *μ*L was used for injection in the chromatographic system.

### 2.5. Procedures for Method Validation

Validation was performed following US FDA guidelines [34]. System suitability was tested by injecting six consecutive injections using aqueous standard mixture of analytes and ISs at the start of each batch during method validation. The precision (% CV) of system suitability test was found in the range of 0.13 to 0.24% for the retention time and 0.85 to 2.96% for the area response for all the analytes and ISs. System performance was studied by injecting one extracted blank (without analytes and ISs) and one extracted LLOQ sample with ISs at the beginning of each analytical batch. The signal-to-noise ratio for system performance was ≥22 for all the three analytes. Autosampler carryover was evaluated by sequentially injecting extracted blank plasma → upper limit of quantitation (ULOQ) sample → two extracted blank plasma sample → LLOQ sample → extracted blank plasma at the start and end of each batch. Selectivity of the method was assessed for potential matrix interferences in ten batches (6 normal lots of K_3_EDTA, 2 haemolysed, and 2 lipemic) of blank human plasma by extraction and inspection of the resulting chromatograms for interfering peaks.

Linearity of the method was assessed from five, ten-point calibration lines. A quadratic, 1/*x* ^2^, least-squares regression algorithm was tested to plot the peak area ratio (analyte/IS) from multiple reaction monitoring versus concentration. The linear equations were then used to calculate the predicted concentrations in all samples within the analytical runs. The correlation coefficient for each calibration curve must be ≥0.99 for all the analytes. The lowest standard on the calibration line was accepted as the LLOQ, if the analyte response was at least ten times more than that of extracted blank plasma. Reinjection reproducibility for extracted samples was also checked by reinjection of an entire analytical run after storage at 5°C.

Intraday accuracy and precision were evaluated by replicate analysis of plasma samples on the same day. The analytical run consisted of a calibration curve and six replicates of HQC, MQC-1/2, LOQ, and LLOQ samples. The interday accuracy and precision were assessed by analysis of five precision and accuracy batches on three consecutive validation days. The precision (% CV) at each concentration level from the nominal concentration should not be greater than 15%. Similarly, the mean accuracy should be within 85–115%, except for the LLOQ, where it can be within 80–120% of the nominal concentration.

Ion suppression/enhancement effects on the MRM LC-MS/MS sensitivity were evaluated by postcolumn analyte infusion experiment. Briefly, a standard solution containing a mixture of ATV, DRV, and RTV (at MQC-1 level) was infused after column into the mobile phase at 10 *μ*L/min employing infusion pump. Aliquots of 5 *μ*L of extracted control blank plasma sample were then injected into the column and chromatograms were acquired for the analytes.

Extraction recovery of the analytes and ISs from human plasma was evaluated in six replicates by comparing the mean peak area responses of preextraction fortified samples to those of postextraction fortified samples representing 100% recovery. Matrix effect, expressed as matrix factors (MFs), was assessed by comparing the mean area response of post-extraction fortified samples with mean area of solutions prepared in mobile phase solutions (neat standards). IS-normalized MFs (analyte/IS) were calculated to access the variability of the assay due to matrix effects. To evaluate the relative matrix effect in different plasma lots, post-extraction fortified samples were prepared in triplicate at LLOQ concentration and assessed for accuracy (%) and precision (% CV). In order to meet acceptance criteria, the % CV must be ≤15% for the analytes.

Stock solutions of analytes and ISs were checked for short-term stability at room temperature and long-term stability at 5°C. Stability results in plasma were evaluated by measuring the area ratio response (analyte/IS) of stability samples against freshly prepared comparison standards with identical concentration. The solutions were considered stable if the deviation from nominal value was within ±10.0%. Autosampler (wet extract), bench top (at room temperature), and freeze-thaw (at −20°C and −70°C) and long-term stability (at −20°C and −70°C) were performed at LQC and HQC level using six replicates. The stability samples were quantified against freshly prepared quality control samples. Stability data were acceptable if the % CV of the replicate determinations did not exceed 15.0% and the mean accuracy value was within ±15.0% of the nominal value.

Method ruggedness was verified with two batches; the first batch was analyzed on two columns with different batch numbers, while the second batch was analyzed by different analysts who were not part of method validation. The ability to dilute samples which could be above the upper limit of the calibration range was validated by analyzing six replicates samples containing 20000/20000/1000 ng/mL of ATV/DRV/RTV after five-/tenfold dilution, respectively. The precision and accuracy for dilution reliability was determined by comparing the samples against freshly prepared calibration curve standards.

## 3. Results

### 3.1. Autosampler Carryover, Linearity, Accuracy and Precision, Limit of Detection, and Limit of Quantitation

**Figure 1:**
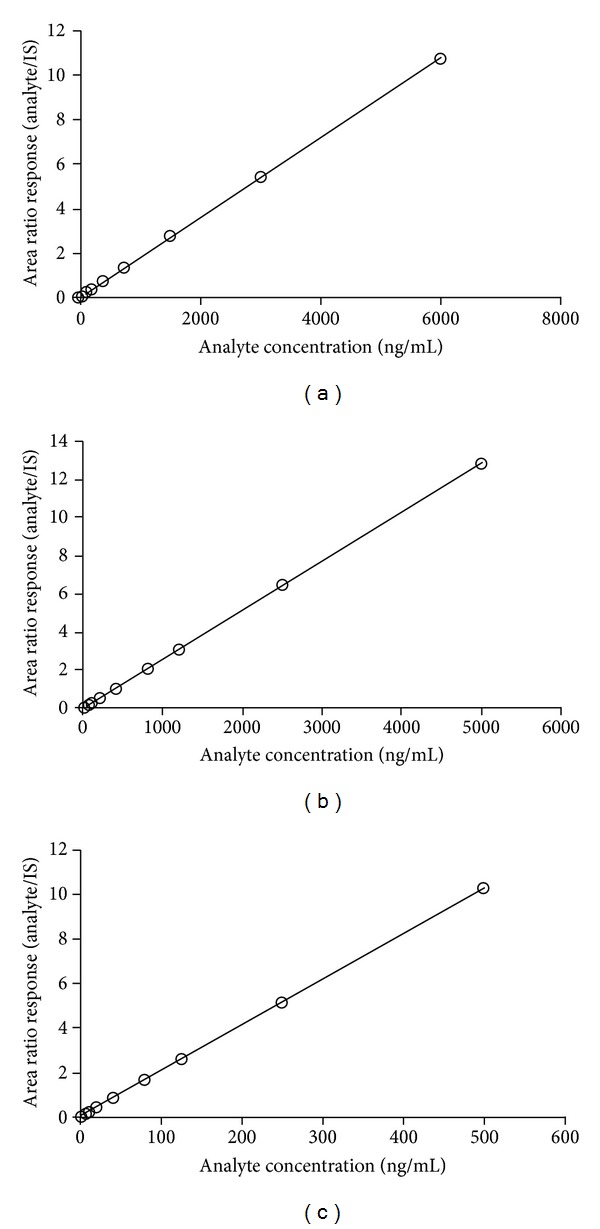
Calibration curves for (a) atazanavir, (b) darunavir, and (c) ritonavir.

The autosampler carryover results showed minimal carryover of analyte, ≤0.12% of LLOQ area in the extracted blank sample after injection of ULOQ sample for the analytes. The calibration curves were linear over the concentration range of 5.0–6000 ng/mL for ATV, 5.0–5000 ng/mL for DRV, and 1.0–500 ng/mL for RTV with a correlation coefficient (*r* ^2^) ≥0.9995 for all the analytes (Figure 1). The mean linear equations obtained were as follows: ATV: *y* = (0.0018 ± 0.0002)*x* + (0.0007 ± 0.0003), DRV: *y* = (0.0025 ± 0.0003)*x* + (0.0002 ± 0.0001), and RTV: *y* = (0.0211 ± 0.0020)*x* + (0.0021 ± 0.0002). The accuracy and precision (% CV) for the calibration curve standards ranged from 95.67 to 105.33% and from 2.19 to 6.34 for ATV, from 93.70 to 103.00% and from 1.68 to 5.66 for DRV and from 98.28 to 103.33% and from 0.61 to 5.92 for RTV. The limit of detection (LOD) and lower limit of quantitation (LLOQ) were 1.5 and 5.0 ng/mL for ATV and DRV and 0.35 and 1.0 ng/mL for RTV respectively. The signal-to-noise ratio for ATV, DRV and RTV was 22 : 1 at LLOQ and 10 : 1 at LOD respectively.

### 3.2. Intra- and Interbatch Accuracy and Precision, Extraction Recovery and Matrix Effect

The intrabatch and interbatch precision (% CV) across five quality control samples ranged from 0.8 to 7.3 over the analytical range and the accuracy was from 91.3 to 104.4% for all the analytes (Table 2). The extraction recovery and matrix factors for the analytes are presented in Tables 3 and 4, respectively. The mean extraction recovery ranged from 97.35 to 101.06 for ATV, from 97.73 to 102.30% for DRV, and from 98.37 to 102.12% for RTV across QC levels. The presence of unmonitored and coeluting compounds from the matrix can affect the accuracy, precision, and overall reliability of a validated method. It is recommended that evaluation of matrix factor (MF) can help to assess the matrix effect. Further, matrix effect needs to be checked in lipemic and haemolysed plasma samples in addition to normal K_3_EDTA plasma. The IS-normalized MFs using stable-isotope labelled IS should be close to unity because of the similarities in the chemical properties and elution times for the analytes and ISs. The IS-normalized MFs ranged from 0.99 to1.03 for all the analytes.

The relative matrix effect was also evaluated in six independent plasma lots which consisted of four normal K_3_EDTA, one haemolysed and one lipemic plasma at LLOQ level. The accuracy and precision values for all the analytes varied from 98.82 to 100.86% and from 1.76 to 3.82%, respectively (Table 5).

### 3.3. Analyte Stability, Method Ruggedness, and Dilution Reliability

The short-term and long-term stability of stock solutions of analytes and ISs were stable at room temperature for up to 7 h and for a minimum period of 7 days, respectively. The stability of all the analytes in plasma was established at appropriate temperatures and storage periods required for clinical analysis. The detailed results for bench top, wet extract, and freeze-thaw and long-term stability of the analytes are summarized in Table 6. The precision and accuracy values observed for method ruggedness (for different columns and analysts) were between 3.5 and 7.6% and between 92.7 and 105.9% for ATV, between 2.5 and 8.6% and between 97.7 and 102.9% for DRV and between 2.6 and 7.9% and between 99.1 and 104.4% for RTV respectively. The dilution integrity experiment was performed with an aim of validating the dilution test to be carried out on higher analyte concentration above ULOQ, which could be found in clinical samples. The precision and accuracy values for 1/5th and 1/10th dilution ranged from 5.0 to 5.6% and from 102.1 to 105.1% for all the analytes.

## 4. Discussion

**Figure 2:**
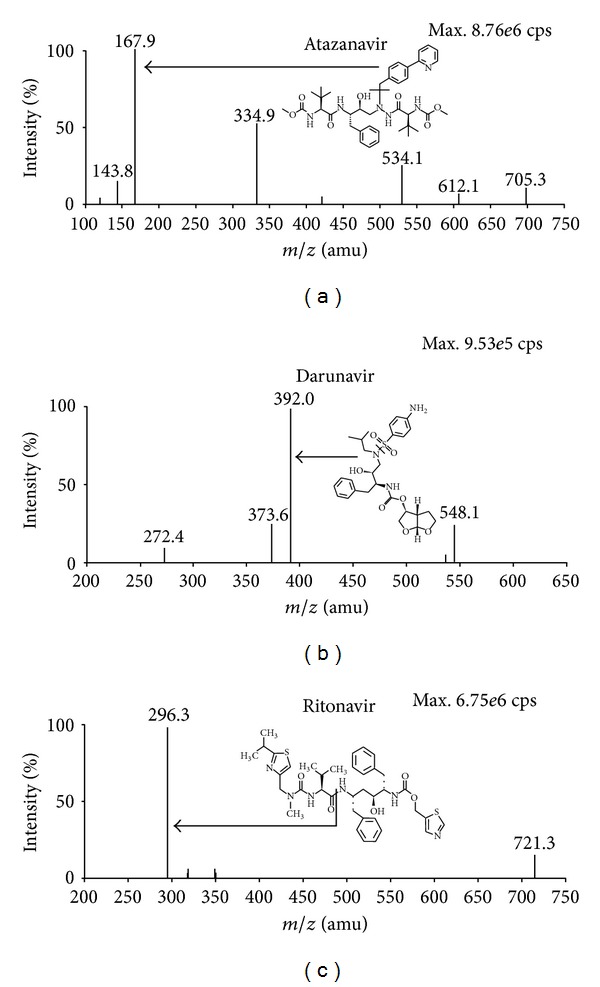
Product ion mass spectra of (a) atazanavir (*m/z* 705.3 → 167.9, scan range 100–750 amu) (b) darunavir (*m/z* 548.1 → 392.0, scan range 200–650 amu), and (c) ritonavir (*m/z* 721.3 → 296.3, scan range 200–750 amu) in the positive ionization mode.

*Method Development.* The present work was executed using electrospray ionization (ESI) in the positive ionization mode as ATV, DRV, and RTV have several secondary amino groups which can be readily protonated. Q1 mass spectra of ATV, DRV, RTV, ATV-d6, DRV-d9, and RTV-d6 contained protonated precursor [M+H]^+^ ions at *m/z* 705.2, 548.1, 721.3, 711.2, 557.1 and 727.4 respectively as reported in our previous work [13, 17, 21]. The most abundant and consistent product ions in Q3 mass spectra for ATV, DRV and RTV were observed at *m/z* 167.9, 392.0 and 296.3 by applying collision energy of 44, 17 and 20 eV respectively. These product ion fragments can be attributed to the substructure 4-(pyridin-2-yl)phenyl methyl group in ATV (Figure 2(a)), elimination of *p*-aminophenyl sulfonyl group from the precursor ion of DRV (Figure 2(b)), and breaking of amide linkage in RTV (Figure 2(c)) respectively. All mass parameters were suitably optimized to obtain a stable and adequate response for the analytes. A dwell time of 200 ms was sufficient and no interference was observed between the MRMs of the analytes and their deuterated ISs.

Methods which deal with the simultaneous determination of these three PIs in human plasma have used protein precipitation (PP) as the extraction technique [22, 25, 35]. Others which deal with simultaneous determination of plasma ATV and RTV [36–39] or DRV and RTV [27] together with other ARVs have employed either PP or liquid-liquid extraction (LLE). Notari et al. [40] determined 16 anti-HIV drugs in human plasma by HPLC using solid-phase extraction (SPE). In our earlier work with ATV [13] and RTV [21], SPE was carried out for their separate determination, while LLE with methyl *tert*-butyl ether was used for DRV [17]. Furthermore, an extensive study was carried to optimize the extraction procedure due to matrix interference during PP and LLE for selective determination of ATV from human plasma [13]. In the present work, SPE was tested on Oasis HLB cartridge for their simultaneous determination in human plasma. Addition of 0.1% formic acid helped in breaking drug-protein binding, with quantitative and precise recovery for the analytes at all QC levels from 50 *μ*L plasma. The plasma volume used for processing is much less compared to reported procedures for simultaneous determination of PIs [20, 22, 25, 36–40].

**Figure 3:**
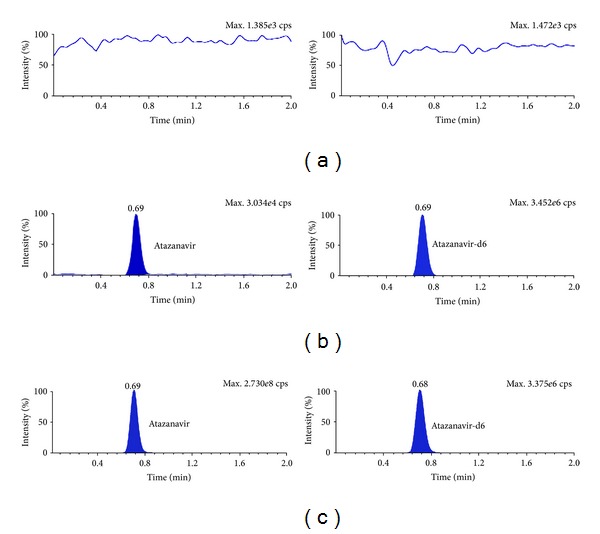
MRM ion chromatograms of atazanavir in (a) double blank plasma (without analyte and IS), (b) at LLOQ and atazanavir-d6, and (c) in real subject sample.

**Figure 4:**
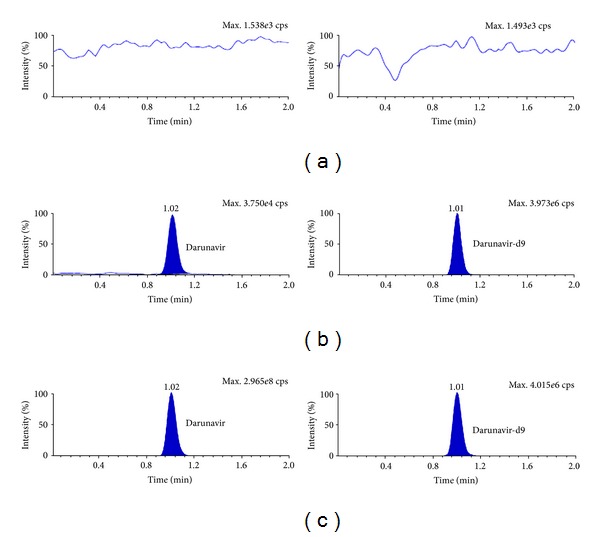
MRM ion chromatograms of darunavir in (a) double blank plasma (without analyte and IS), (b) at LLOQ and darunavir-d9, and (c) in real subject sample.

**Figure 5:**
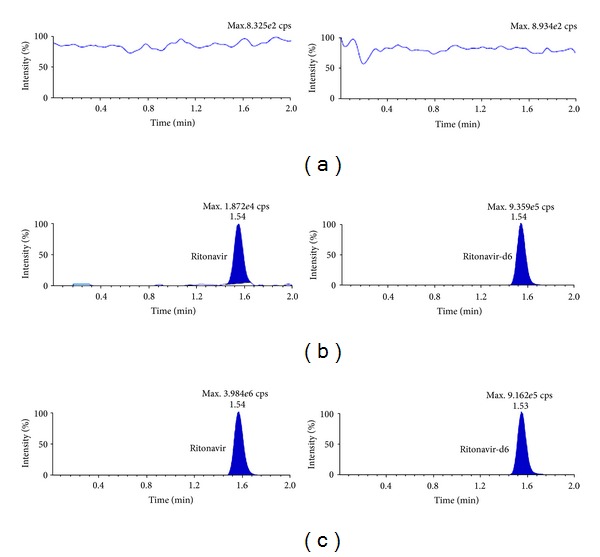
MRM ion chromatograms of ritonavir in (a) double blank plasma (without analyte and IS), (b) at LLOQ and ritonavir-d6, and (c) in real subject sample.

The chromatographic conditions were initiated to have short run time, adequate response and good peak shapes under isocratic conditions on Waters Acquity UPLC BEH C18 (50  ×  2.1 mm, 1.7 *μ*m) column. Based on our earlier work for ATV and RTV [13, 21], various combinations of organic solvents (methanol/acetonitrile) together with ammonium formate/formic acid buffer in the pH range 3.5–5.5 were tried. However, the run time was more than 4.0 min for baseline resolution of the analytes. Thus, gradient elution was tried using ammonium formate and acetonitrile, and the best mobile phase conditions were obtained using solvent system (A) 10 mM ammonium formate, pH 4.0 adjusted with formic acid and (B) acetonitrile to achieve adequate retention, peak shape, adequate response and complete separation. All the analytes were eluted within 2.0 min with retention time of 0.69, 1.02 and 1.54 for ATV, DRV and RTV respectively. Further, the reproducibility of retention times for the analytes, expressed as % CV was ≤0.52% for 100 injections on the same column. The capacity factors and number of theoretical plates which are used to characterize the performance of chromatography are summarized in Table 1. The resolution factor (*R*_*s*_) between ATV and DRV and DRV and RTV was 2.06 and 3.25 respectively. Further, use of deuterated internal standards helped to compensate any variability during extraction and UPLC-MS/MS analysis. MRM chromatograms for double blank plasma (without analyte and IS), at LLOQ and a real subject sample in Figures 3, 4 and 5 confirm the selectivity of the method to distinguish and quantify the analyte from endogenous components in the plasma matrix. Moreover, there was no interference of matrix at the retention time of analytes or ISs as evident from postcolumn infusion study.

## 5. Conclusions

In spite of several existing assay methods for the simultaneous determination of PIs, very few studies have reported the use of UPLC-MS/MS for therapeutic drug monitoring. In this present work, we have developed and fully validated a reliable, precise and sensitive UPLC-MS/MS method for the simultaneous quantification of atazanavir, darunavir and ritonavir in human plasma. The assay is superior to reported methods with respect to sensitivity, analysis time and matrix effect. The method is rapid and requires small plasma volume for sample processing. Use of deuterated internal standards further reinforces the accuracy and precision of the proposed method and can be suitable for pharmacokinetic/bioequivalence studies.

## Figures and Tables

**Table 1:** Optimized mass spectrometer parameters, MRM transitions, and chromatographic performance.

Parameters	ATV	ATV-d6	DRV	DRV-d9	RTV	RTV-d6
Mass spectrometry parameters						

Source dependent						
Capillary voltage (kV)			4.0			
Extractor voltage (V)			3.0			
RF lens (V)			0.0			
Source temperature (°C)			110			
Desolvation temperature (°C)			400			
Desolvation gas flow (L/h)			900 ± 10			
Cone gas flow (L/h)			100 ± 10			
Analyzer parameters						
LM 1/HM 1 resolution			15.0/15.0			
Ion energy 1/ion energy 2			0.2/1.0			
Entrance/exit			−1.0/0.1			
LM 2/HM 2 resolution			14.0/14.0			

Compound dependent						
Cone voltage (V)	30	29	25	27	30	26
Collision energy (eV)	44	42	17	17	20	21
Dwell time (ms)	200	200	200	200	200	200
MRM transition (*m/z*)	705.3/167.9	711.2/168.0	548.1/392.0	557.1/401.0	721.3/296.3	727.4/302.3

Chromatography characteristics						

Retention time (min)	0.69	0.69	1.02	1.01	1.54	1.54
Capacity factors	1.15	1.16	2.18	2.15	3.81	3.82
Theoretical plates	396	398	851	836	1683	1682

ATV: atazanavir; ATV-d6: atazanavir-d6; DRV: darunavir; DRV-d9: darunavir-d9; RTV: ritonavir; RTV-d6: ritonavir-d6.

RF: radio frequency; LM: low mass; HM: high mass.

**Table 2:** Intrabatch and interbatch precision and accuracy for atazanavir, darunavir, and ritonavir.

QC level (nominal concentration)	Intrabatch (*n* = 6; single batch)			Interbatch (*n* = 30; 6 from each batch)		
	Mean concentration observed (ng/mL)	% CV	% Accuracy	Mean concentration found for 5 batches (ng/mL)	% CV	% Accuracy
Atazanavir						
LLOQ QC (5.0 ng/mL)	5.21	6.3	104.1	5.05	5.1	101.0
LQC (15 ng/mL)	14.5	4.5	96.8	14.9	1.5	99.4
MQC-2 (150 ng/mL)	142.8	2.8	95.2	153.9	4.3	102.6
MQC-1 (2400 ng/mL)	2362	3.4	98.4	2482	0.8	103.4
HQC (4800 ng/mL)	4886	3.3	101.8	4814	3.2	100.3

Darunavir						
LLOQ QC (5.0 ng/mL)	4.89	6.9	97.8	4.97	7.3	99.5
LQC (15 ng/mL)	14.3	4.1	95.3	14.7	3.2	98.1
MQC-2 (150 ng/mL)	149.5	1.9	99.7	153.0	1.4	102.0
MQC-1 (2000 ng/mL)	2088	3.0	104.4	1936	3.5	96.8
HQC (4000 ng/mL)	3832	2.3	95.8	3808	1.4	95.2

Ritonavir						
LLOQ QC (1.0 ng/mL)	0.95	5.8	95.0	0.93	5.8	92.7
LQC (3.0 ng/mL)	2.78	4.9	92.7	2.86	1.3	95.2
MQC-2 (30 ng/mL)	30.7	1.9	102.2	30.9	2.9	103.2
MQC-1 (200 ng/mL)	182.6	3.8	91.3	189.2	4.4	94.6
HQC (400 ng/mL)	385.6	4.9	96.4	404.4	1.9	101.1

CV: coefficient of variation; LLOQ: lower limit of quantitation; LQC: low quality control; MQC: medium quality control; HQC: high quality control.

**Table 3:** Extraction recovery of atazanavir, darunavir, and ritonavir from human plasma.

QC level	Atazanavir			Darunavir			Ritonavir		
	Area response		Extraction recovery, % (*B*/*A*)	Area response		Extraction recovery, % (*B*/*A*)	Area response		Extraction recovery, % (*B*/*A*)
	*A *	*B *		*A *	*B *		* A *	*B *	
LQC	9773	9514	97.35	20074	19861	98.94	6074	6118	100.72
MQC-2	104201	102458	98.33	183148	187367	102.30	64148	63283	98.65
MQC-1	1654728	1694608	101.06	2491752	2435246	97.73	390617	384267	98.37
HQC	3382518	3408890	100.16	4922563	4847816	98.48	821547	838931	102.12

QC level	Atazanavir-d6			Darunavir-d9			Ritonavir-d6		
	Area response		Extraction recovery, % (*B*/*A*)	Area response		Extraction recovery, % (*B*/*A*)	Area response		Extraction recovery, % (*B*/*A*)
	* A *	*B *		* A *	*B *		* A *	*B *	

LQC	338514	335727	99.18	530681	531079	100.07	105065	103329	98.35
MQC-2	348240	339419	97.47	510151	525715	103.05	102738	102443	99.71
MQC-1	341729	346341	101.35	541747	535298	98.81	98827	96357	97.50
HQC	331508	337897	101.93	520571	517827	99.47	97538	99945	102.47

LQC: low quality control; MQC: medium quality control; HQC: high quality control.

*A*: mean area response of six replicate samples prepared by spiking in extracted blank plasma.

*B*: mean area response of six replicate samples prepared by extracting spiked blank plasma.

**Table 4:** Matrix factor for atazanavir, darunavir, and ritonavir.

QC level	Atazanavir			Darunavir			Ritonavir		
	Area response		Matrix factor (*B*/*A*)	Area response		Matrix factor (*B*/*A*)	Area response		Matrix factor (*B*/*A*)
	*A *	*B *		*A *	*B *		*A *	*B *	
LQC	10061	9773	0.97	19583	20074	1.03	6137	6074	0.99
MQC-2	103108	104201	1.01	189378	183148	0.97	63019	64148	1.02
MQC-1	1694608	1654728	0.98	2487277	2491752	1.00	404237	390617	0.97
HQC	3408890	3382518	0.99	5047023	4922563	0.98	846102	821547	0.97

QC level	Atazanavir-d6			Darunavir-d9			Ritonavir-d6		
	Area response		Matrix factor (*B*/*A*)	Area response		Matrix factor (*B*/*A*)	Area response		Matrix factor (*B*/*A*)
	*A *	*B *		*A *	*B *		*A *	*B *	

LQC	347253	338514	0.97	531482	530681	1.00	103941	105065	1.01
MQC-2	344253	348240	1.01	523378	510151	0.97	104106	102738	0.99
MQC-1	346748	341729	0.99	534156	541747	1.01	100574	98827	0.98
HQC	340675	331508	0.97	532074	520571	0.98	100078	97538	0.97

LQC: low quality control; MQC: medium quality control; HQC: high quality control.

*A*: mean area response of six replicate samples prepared in mobile phase (neat samples).

*B*: mean area response of six replicate samples prepared by spiking in extracted blank plasma.

**Table 5:** Relative matrix effect in different lots of human plasma at LLOQ level.

Analyte (nominal concentration)	Mean area response in six plasma lots (mean of three replicates)						Coefficient of variation (%)	Accuracy (%)
	(1) K_3_EDTA	(2) K_3_EDTA	(3) K_3_EDTA	(4) K_3_EDTA	(5) Haemolysed	(6) Lipemic		
Atazanavir (5.0 ng/mL)	3415	3368	3418	3497	3625	3707	3.82	100.86
Darunavir (5.0 ng/mL)	6245	6351	6471	6152	6283	6373	1.76	98.82
Ritonavir (1.0 ng/mL)	2004	2048	2105	2067	2117	2037	2.06	99.23

LLOQ: lower limit of quantitation.

**Table 6:** Stability of atazanavir, darunavir, and ritonavir in human plasma under different conditions.

Storage conditions	Atazanavir		Darunavir		Ritonavir	
	Mean of six stability samples (ng/mL) ± SD	% change	Mean of six stability samples (ng/mL) ± SD	% change	Mean of six stability samples (ng/mL) ± SD	% change
Bench top stability at ambient temperature; 14 h						
LQC	15.23 ± 0.20	1.33	15.41 ± 0.44	2.73	2.954 ± 0.189	−1.53
HQC	4885 ± 155	1.78	4215 ± 215	5.38	417.1 ± 24.65	4.28

Wet extract stability; 24 h, 5°C						
LQC	15.42 ± 0.32	2.67	15.24 ± 0.94	1.60	3.068 ± 0.135	2.27
HQC	4893 ± 267	1.94	3975 ± 176	−0.63	418.2 ± 16.75	4.55

Freeze and thaw stability in plasma; 6 cycles, −20°C						
LQC	14.74 ± 0.28	−2.11	15.71 ± 0.76	4.73	2.865 ± 0.143	−4.50
HQC	4717 ± 149	−1.73	4058 ± 138	1.45	415.6 ± 21.67	−3.65

Freeze and thaw stability in plasma; 6 cycles, −70°C						
LQC	14.96 ± 0.51	−0.67	14.85 ± 0.82	−1.00	2.981 ± 0.176	−0.63
HQC	4687 ± 178	−2.35	4156 ± 202	3.90	378.6 ± 14.39	3.90

Long-term stability in plasma; 60 days, −20°C						
LQC	15.31 ± 0.43	2.02	15.28 ± 0.79	1.87	3.073 ± 0.159	2.43
HQC	4924 ± 231	2.58	4187 ± 237	4.68	378.6 ± 17.83	−5.35

Long-term stability in plasma; 60 days, −70°C						
LQC	15.53 ± 0.27	3.34	14.98 ± 0.56	−0.13	3.043 ± 0.213	1.43
HQC	5032 ± 97	4.83	3947 ± 191	−1.33	374.9 ± 13.74	−6.28

LQC: low quality control; HQC: high quality control.

SD: standard deviation; *n*: number of replicates at each level.

% change = ((mean stability samples – mean comparison samples)/mean comparison samples) × 100.
